# The Influence of the Social Environment Context in Stress and Coping in Sport

**DOI:** 10.3389/fpsyg.2016.00875

**Published:** 2016-06-14

**Authors:** Carlijn Kerdijk, John van der Kamp, Remco Polman

**Affiliations:** ^1^Faculty of Human Movement Science, Vrije UniversiteitAmsterdam, Netherlands; ^2^Center for Behavior Change, Psychology Department, Bournemouth UniversityPoole, UK

**Keywords:** stress, coping, social environment, sport, appraisal

## Abstract

Lazarus (1999) model of stress and coping is based on the reciprocal interaction between the person and the environment. The aim of this study therefore was to examine whether the social environment (significant others) are of influence on the stress and coping of team athletes. The study consisted of two separate studies in which a total of 12 team athletes participated. First, six field hockey players (two males, four females) aged 18–29 years (*M* = 23.0 years) participated in a diary study. Second, six team athletes of different sports (two males, four females) aged 24–29 years (*M* = 25.8 years) were interviewed. The results showed that in particular teammates are important for the appraisal of stress and coping in team sports. For over half (i.e., 51.5%) of the reported stressors in the diary study the participants felt that others were of influence on their coping. Team athletes experienced the highest stress intensity during competition, or when they appraised the situation as a threat. When others were of influence the team athletes were most likely to appraise the situation as a challenge and use problem- or emotion-focused coping strategies. These finding might provide a new portal for intervention to enhance coping with stress in sport and enhance performance and satisfaction.

## Introduction

The most widely used model to understand the relation between stress, coping, and emotions (Nicholls and Polman, 2007) is the transactional model (Lazarus, 1999). This model explains the relation between stress and coping as a dynamic process concerning the individual’s internal and situational environment, where the person appraises the situation through primary and secondary appraisal (Lazarus, 1999). Primary appraisal is the person’s belief of the significance of the situation related to their personal values, beliefs or intentions. Secondary appraisal refers to a complex evaluative process examining the coping options available to limit negative and increase positive outcomes (Lazarus and Folkman, 1984).

Athletes have multiple options to cope with stressful situations and minimize potential harm. Problem-focused coping strategies reduce the impact of the stressor by focussing on a task or action (Lazarus and Folkman, 1984). Regulation of emotions that are incited by a stressor is considered as an emotion-focused coping strategy (Lazarus and Folkman, 1984). The athlete uses an avoidance coping strategy when he/she is trying to keep away from the stressor, by either cognitively (e.g., blocking thoughts) or behaviourally (e.g., walking away from the stressor) avoiding the stressor (Krohne, 1993).

The coping strategies used by athletes are influenced by whether they perceive a stressful encounter as a threat or a challenge (Anshel et al., 2001; Levy et al., 2010). Threat appraisal is associated with more frequent use of maladaptive coping strategies, negative emotions and lower self-rated performance scores. Challenge appraisal, on the other hand, results in the use of adaptive coping strategies, positive emotions higher self-rated performance (Nicholls et al., 2012).

**FIGURE 1:**
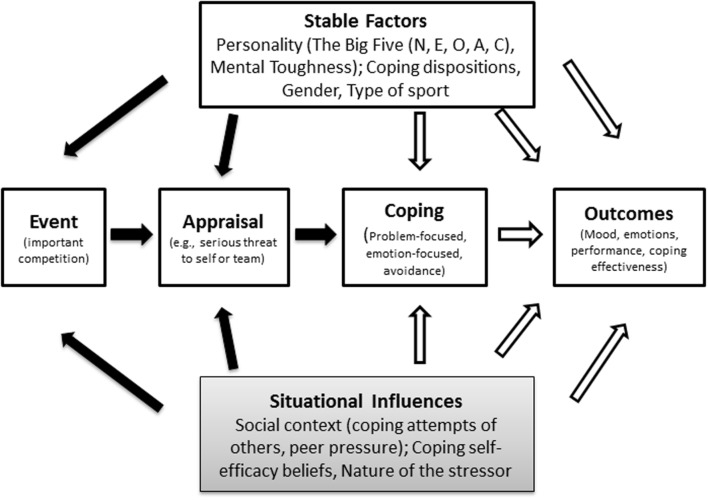
**Conceptual framework of how stable factors and situational factors can directly and indirectly influence the stress and coping process in the domain of sport**.

Based on DeLongis and Holtzman (2005) **Figure 1** provides an overview of previous research which has examined possible direct and indirect effects of stable factors and situational influences on the stress and coping process in sport. Previous research has examined direct and indirect effects of stable factors on the stress and coping process in sport. For example, personality (Kaiseler et al., 2012a), gender (Kaiseler et al., 2012b), and type of sport (Giacobbi et al., 2004) have been shown to influence how athletes appraise stressful events and the way they cope. However, situational factors also influence the stress and coping process and are defined as ‘related to the immediate nature of the stressful transaction, which was the specific focus of the individual’s coping attempts (Parkes, 1986, p. 1279).

Some research has examined situational factors including the role of sources of stress (Anshel and Kaissidis, 1997) and perceptions of control (Kaiseler et al., 2012b) on the stress and coping process. Research on game location/home advantage in sport provide indirect evidence that the social context might influence stress and coping in sport. This research has shown that those competing at home in team and individual sports are more likely to be successful (Carron et al., 2005) with research in basketball suggesting that in particular away players engage in more dysfunctional assertive behaviors (e.g., more fouls; McGuire et al., 1992). We conducted a systematic review for studies examining the role of the social context and in particular the role of significant other persons on the stress and coping process. No relevant study was found.

We have defined the role of significant others on stress and coping as *‘the experienced influence of the uncalled-for behavior (verbal or non-verbal) of others on the athlete’s appraisal of stress and their coping.’* This distinguish this influence from social support, the *‘process of interaction in relationships which improves coping, esteem, belonging, and competence through actual or perceived exchanges of physical or psychological resources’* (Gottlieb, 2000, p. 28). Besides highlighting outcomes of social support this definition also stresses the relevance of communication and that social support is an interactive process in which individuals exchange physical or psychological resources. Our definition is also distinct from social support as a coping strategy. Hence, informational or emotional social support coping strategies are conscious efforts of athletes to deal with a stressful encounter.

Previous research into stress and coping has been limited to studies examining the role of person factors. Therefore, this study examined the role of others in team sports on the way athletes experience and appraised stressful encounters and in the way they coped with these stressors. This is, to our knowledge, the first study examining the role of the social environment (i.e., through others) on the stress and coping process and will therefore be inductive in nature. We therefore did not make any specific predictions. To address the aim of the study, two different methods were used. We used a diary method to collect data over time thereby reducing the effects of retrospective recall of a sample of field hockey players (Nicholls et al., 2006). Secondly, the interview method was used to obtain more in-depth account of stress and coping in a social context. This was done with a sample of athletes from different team sports to explore whether findings could be generalized across sports.

## Materials and Methods

### Study 1: Diary Study

#### Participants

The participants for the diary part of this research were six elite field hockey players (two males, four females), aged 18–29 years (*M* = 23.0 years, *SD* = 4.2). Their sport experience varied between 12 and 20 years (*M* = 16.2 years, *SD* = 3.2). The level in which the participants played was the highest or second highest hockey competition league in The Netherlands or Belgium. The study had the approval of a University ethics committee in Australia and The Netherlands. All participants provided consent before the start of the research.

#### Procedure

In both studies the influence of others was defined as *the experienced influence of unsolicited behavior (verbal or non-verbal) of others on the participants’ appraisal of stress and their coping*.

To assess stress and coping longitudinally an online diary was developed. Participants were required to complete the diary on each day they engaged in a training session or competition during a period of 21 days. The diary was completed on a personal computer as soon as possible following a training session or competition. To improve adherence participants were contacted by email on each competition or training day (Nicholls et al., 2009).

#### Instruments

The diary consisted of an online form in which participants answered seven questions. Firstly, they were asked to list ‘the demands you encountered that caused worry or negative emotions’ (Aldwin, 2007). Participants were asked if the situation was perceived to be a challenge or threat and to ‘rate the intensity of your feeling after the stressor occurred.’ Stress intensity was scored on a 5 point Likert scale where 1 = *‘not intense’* and 5 = *‘extremely intense.’* Regarding the coping strategies, an open-ended format was used whereby participants were asked for each stressor to ‘write down what you did to manage the stressor.’ Following this the participants were asked whether the stressor and coping response was influenced by others (answering with yes or no), and when yes to ‘describe how others influenced your reaction to the event and the way you coped.’

#### Data Analysis

The written open-ended stressors and coping responses from the diary study were transcribed verbatim and subjected to an inductive and deductive content analysis procedure (Maykut and Morehouse, 1994). Similar stressors and coping strategies were identified and assigned a descriptive label and a general rule of inclusion was written. Once these themes were created and the data analysis procedure progressed, stressors and coping strategies that appeared to fit into an established category were deductively analyzed within that particular theme (Patton, 2002). Coping was analyzed at the dimensional level. Therefore, coping strategies identified were categorized into one of four dimensions: Problem-focussed coping, emotion-focussed coping, avoidance coping or no coping. Data was analyzed by the first author and a 10% of the sample was analyzed by the third author. Following discussion they achieved 100% agreement.

Stressor intensity in relation to threat/challenge and training sessions and competitions were calculated, and then divided by their frequency. This generated a mean intensity rating for each stressor when appraised as either a threat or a challenge during either training or competition. Student *t*-tests were performed to examine differences in stress intensity during game or training, when stress was appraised as a threat or challenge, and when there was or was not felt the influence of others on stress and coping during the situation. A one-way ANOVA was conducted to see whether there was a difference between the experienced stress intensity between the four higher order coping dimensions. The calculation of a χ^2^ for the four higher order coping dimensions and the influence of others were performed to examine whether there was a difference between chosen coping dimension and the influence of others.

## Results Study 1

The participants completed 81.4% of their diary entries in a timely manner over the 21 days diary period. All 28 games of the participants were noted in their diaries (100%). The participants completed 31 of the 44 training session entries which were planned over the 21 days diary period (70.5%). Missing training entries were related to injury, canceled training sessions through bad weather, or off field team activities.

### Stressors

The frequencies and percentage, as a function of situation (training or competition), type and influence of others, which were recorded in the diaries, are shown in **Table 1**. More stressors were reported during competition and slightly more stressors were appraised as a threat (54.4%) than as a challenge. In 51.5% of the reported situations the athletes indicated that others had an influence. There was no significant difference found between the frequencies of the different stressors when it was appraised that others were of influence and where others were not appraised as an influence (χ^2^ = 3.73; *P* = 0.81). Visual inspection of the data indicates that the highest reported frequency of others being of influence on the coping of the participants was related to the official (22.9%).

**Table 1:** Frequency of stressors experienced by the athletes during competition and training, whether appraised as a challenge or a threat and whether influenced by others or not.

	Situation		Type		Influence of Others		Total (*n* = 68)
Stressors	Competition (*n* = 36)	Training (*n* = 32)	Threat (*n* = 37)	Challenge (*n* = 31)	Yes (*n* = 35)	No (*n* = 33)	
Official	10 (27.8%) (*M* = 3.9)	2 (6.3%) (*M* = 2.5)	9 (24.3%) (*M* = 4.2)	3 (9.7%) (*M* = 2.3)	8 (22.9%) (*M* = 3.9)	4 (12.1) (*M* = 3.3)	12 (17.6%) (*M* = 3.7)
Performance/ Outcome	5 (13.9%) (*M* = 3.2)	4 (12.5%) (*M* = 2.8)	3 (8.1%) (*M* = 4.3)	6 (19.4%) (*M* = 2.3)	5 (14.3%) (*M* = 3.6)	4 (12.1%) (*M* = 2.3)	9 (13.2%) (*M* = 3.0)
Team-mate	8 (22.2%) (*M* = 3.4)	9 (28.1%) (*M* = 2.9)	11 (29.7%) (*M* = 3.3)	6 (19.4%) (*M* = 3.0)	7 (20.0%) (*M* = 3.6)	10 (30.3%) (*M* = 3.3)	17 (25.0%) (*M* = 3.2)
Injury	1 (2.8%) (*M* = 5.0)	3 (9.4%) (*M* = 3.7)	4 (10.8%) (*M* = 4.0)	–	2 (5.7%) (*M* = 5.0)	2 (6.1%) (*M* = 3.0)	4 (5.9%) (*M* = 4.0)
Opponent	2 (5.6%) (*M* = 3.5)	1 (3.1%) (*M* = 4.0)	–	3 (9.7%) (*M* = 3.7)	1 (2.9%) (*M* = 4.0)	2 (6.1%) (*M* = 3.5)	3 (4.4%) (*M* = 3.7)
Coach	2 (5.6%) (*M* = 4.0)	2 (6.3%) (*M* = 1.5)	1 (2.7%) (*M* = 4.0)	3 (9.7%) (*M* = 2.3)	1 (2.9%) (*M* = 2.0)	3 (9.1%) (*M* = 3.0)	4 (5.9%) (*M* = 2.8)
Error	3 (8.3%) (*M* = 3.3)	4 (12.5%) (*M* = 3.5)	3 (8.1%) (*M* = 3.7)	4 (12.9%) (*M* = 3.3)	4 (11.4%) (*M* = 3.5)	3 (9.1%) (*M* = 3.3)	7 (10.3%) (*M* = 3.4)
Miscellaneous	5 (13.9%) (*M* = 4.0)	7 (21.9%) (*M* = 2.9)	6 (16.2%) (*M* = 3.8)	6 (19.4%) (*M* = 2.7)	7 (20.0%) (*M* = 3.4)	5 (15.2%) (*M* = 3.0)	12 (17.6%) (*M* = 3.3)
Total	36 (53.0%) (*M* = 3.7)	32 (47.0%) (*M* = 3.0)	37 (54.4%) (*M* = 3.8)	31 (45.6%) (*M* = 2.8)	35 (51.5%) (*M* = 3.5)	33 (48.5%) (*M* = 3.1)	68 (100%) (*M* = 3.3)

*Also reported the mean stress intensity reported by the participants.*

A *t*-test showed a difference in the stress intensity experienced in a competition or training situation (*t*_60_ = 2.42; *P* = 0.02; *d* = 0.59) and in a threat or challenge situation (*t*_59_ = 3.98; *P* < 0.001; *d* = 0.98). The participants experienced higher stress intensity during competition situations (*M* = 3.7, *SD* = 1.0) compared to training situations (*M* = 3.0, *SD* = 1.2) and during threat situations (*M* = 3.8, *SD* = 0.09) compared to challenge situations (*M* = 2.8, *SD* = 1.1). Finally, whether the athletes felt the influence of others (*M* = 3.5, *SD* = 1.1) or did not feel influenced by others (*M* = 3.1, *SD* = 1.2), did not make a significant difference for the experienced stress intensity of the participants (*t*_65_ = 1.65; *P* = 0.09; *d* = 0.40).

### Coping

**Table 2** shows the frequencies and percentage of the coping dimensions reported by the participants. Of the 68 coping strategies reported, 28 strategies were classified as problem-focused coping (41.2%), 13 as emotion-focused coping (19.1%), 11 as avoidance coping (16.2%) and in 16 situations (23.5%) no explicit coping strategy was employed. Problem-focussed coping strategies included problem solving (11.8%), information seeking (2.9%), increasing effort (13.2%), and communication (13.2%). Emotion-focused coping consisted of the strategies relaxation (1.5%), self-blame (2.9%), acceptance (1.5%), wishful thinking (1.5%), positive orientation (5.9%), visualization (1.5%), venting emotions (4.4%). Finally, blocking (8.8%) and behavioral avoidance (7.4%) where the avoidance coping strategies reported.

**Table 2:** Frequency of coping by dimension for competition and training, whether situation was viewed as a threat or challenge or influenced by others.

	Situation		Type		Influence of Others		Total (*n* = 68)
Coping dimension	Competition (*n* = 36)	Training (*n* = 32)	Threat (*n* = 37)	Challenge (*n* = 31)	Yes (*n* = 35)	No (*n* = 33)	
Problem-focused	19 (52.8%)	9 (28.1%)	12 (35.1%)	15 (48.4%)	16 (45.7%)	12 (36.4%)	28 (41.2%)
Emotion-focused	8 (22.2%)	5 (15.6%)	5 (13.5%)	8 (25.8%)	9 (25.7%)	4 (12.1%)	13 (19.1%)
Avoidance coping	3 (8.3%)	8 (25.0%)	8 (21.6%)	3 (9.7%)	4 (11.4%)	7 (21.2%)	11 (16.2%)
No Coping	6 (16.7%)	10 (31.3%)	11 (29.8%)	5 (16.1%)	6 (17.1%)	10 (30.3%)	16 (23.5%)
Total	36 (53.0%)	32 (47.0%)	37 (54.4%)	31 (45.6%)	35 (51.5%)	33 (48.5%)	68 (100%)

The data showed that in competition situations more problem- (52.8%) and emotion-focused coping strategies (22.2%) were reported by the participants, while in training situations avoidance coping strategies (25.0%) and no use of coping (31.3%) were more often used to deal with stress. When the participants appraised the stressor as a threat, they mainly used avoidance coping strategies (21.6%) or no coping (29.8%), but when the stressor was appraised as a challenge they more often applied a problem- (48.4%) or emotion-focused coping strategy (25.8%). To assess whether the use of a particular coping dimension was associated with reporting higher stress intensity a one-way ANOVA was conducted. There was no difference in stress intensity between the four coping dimensions [*F*(3,67) = 1.27; *P* = 0.29].

### Influence of others

The participants mentioned 35 situations in which they perceived others influencing their coping. In 32 situations (91.4%) the participants reported that teammates were the ones influencing their coping behavior (1x crowd; 2x coach). The mean stress intensity for situations in which teammates influenced coping behavior was 3.1).

In situations where the participants reported that others were of influence, they reported more frequent use of problem- (45.7%) or emotion-focused coping strategies (25.7%). In contrast, when stressful encounters were not rated as being influenced by others more avoidance coping strategies (21.2%) or no coping (30.3%) was utilized. Also, stressful encounters in which others were said to play a role were more likely to be appraised as a challenge, and were more likely to occur during competitions. In contrast, when it was rated that others were not influencing coping, the situation was more likely to be appraised as a threat and occurred more often during training situations.

### Study 2: Interviews

#### Participants

Six participants (two males, four females), aged 24–29 years (*M* = 25.8 years; *SD* = 2.3) from a variety of sports (soccer, ice-hockey, futsal and cricket) took part in the interview part of this research. Their sport experience varied between 3 and 20 years (*M* = 13.3 years, *SD* = 6.2). All participants provided consent before the start of the research.

#### Procedure

Six team athletes were interviewed face-to-face, within 3 days of their last game, to examine the effect of others on the appraisal of stressors and on the way athletes coped with such situations. Interviews were conducted either in a private room at Victoria University, Melbourne or over Skype with participants in the Netherlands. The semi-structured interviews lasted between 20 and 45 min and only focussed on game situations. The interviews were transcribed verbatim before being analyzed.

An interview guide consisting of open-ended questions was developed. The participants were asked to describe a specific situation in their last game that caused them stress which was influenced by team members or other significant individuals. After the participants described the situation they were asked to describe what they did to cope with the situation and to explain what team members or other significant individuals, in their opinion, did to help or influence the way they coped with the stressor.

#### Data Analysis

The interviews were analyzed using a thematic analysis approach (Braun and Clarke, 2006). The interviews were read and re-read by the first researcher, and during the second reading stressors, feelings and thoughts, coping, and the influence of others was highlighted with different colors. If necessary, notes on the coping or influence of others were written down in the left-hand margin. The highlighted text blocks that referred to coping and the influence of others were extracted and ordered into categories. The third author also analyzed a subsample of the interviews. Following discussion both researchers achieved 100% agreement on the stress and coping categories generated and the statements attributed to these categories.

## Results Study 2

In the interviews, 37 situations were reported in which the stressful encounter and the use of coping strategies was influenced by others. The raw data were coded into six first-order categories, which in turn were collapsed into three second-order categories (see **Table 3**). In the Support category two first-order-categories were identified that were related to Individual support and Team spirit. The most quotes were related to the second-order category Communication, which consisted of three first-order categories: Negative communication and style, Positive communication and style and Mixed message. The result showed that in most cases teammates were the sender of the messages that influenced the stress and coping process. In most cases the athletes valued the way others influenced their coping behavior.

**Table 3:** Results of the qualitative data analysis.

Second order Theme	First order Themes	Raw data
Support	Individual support	They were saying don’t worry about it… I think you are really good in this and so on.
		They were giving me heaps of tips and stuff. Which was pretty good.
	Team spirit	I think it is important to get everyone enjoying that they are out there. As soon as they start to enjoy it out there, the performance seems to take care of itself.
		Having some motivation or positive reinforcement helps. Knowing that in that situation I assisted the team and helped to achieve the goal that everyone wants to achieve.
Effort	Effort	After one of us has spoken and said ‘let’s work on this’
		If people are putting in a lot of effort, you tend to join them in that…
Communication	Negative messages and style	He starts yelling at me and blaming me…
		In a team meeting, two team mates blamed and accused me of things, they made me feel like the black sheep of the team.
	Positive Messages and style	When we talk in games it tends to work better.
		If people start to talk more, more smiles, and a better positive energy.
	Mixed messages	One told me to step left, the other told me to step right. I did what I thought was the best option for that movement.
		No it is more that they just want the best out of you. And they go like you should be doing this or this. Everyone means well, but people might provide help when you don’t really need it.

## Discussion

The findings of our studies show that teammates play an important role in the experience and appraisal of stress and coping in team sports. In approximately 50% of stressful encounters athletes reported that others influenced the way they appraised and coped with the situation but others did not influence stress intensity levels. When others influenced the stress and coping process situations were more likely to be appraised as a challenge and more adaptive problem- and emotion-focussed coping strategies were used. When a stressful encounter was appraised as a threat it was less like to be influenced by others and more maladaptive avoidance coping and no coping was reported. This is the first study that has examined the role the social environment context on the stress and coping process. Both studies indicated that athletes appraisal of the stressful encounter and the way they cope is influenced by the way others and in particular teammates respond. The diary study indicated that this happened in around 50% of all the stressful encounters during both competition and training.

The type of stressors reported and the notion that four stressors (Official, Performance/Outcome, Teammate and Error) accounted for 66.1% of the reported stressors is in line with results of previous longitudinal studies (Nicholls et al., 2006; Gan et al., 2009) in which a small number of stressors reoccurred over time. Previous studies have also indicated that athletes report higher levels of stress intensity during competition compared to training (Nicholls et al., 2009) and when they appraise a situation as a threat in comparison to a challenge (Anshel et al., 2001). This is in line with the findings of the current study where the diary participants reported higher stress intensity during competition and when they appraised the situation as a threat. Interestingly, when the athletes indicated that other had an influence on the stress and coping process they were more likely to appraise the situation as a challenge. However, the influence of others did not influence self-reported stress intensity levels.

The coping strategy an athlete uses is indirectly influenced through the appraisal of the stressor. Results from the diary study show that an athlete is more likely to use an avoidance coping strategy or no coping when the stressor is appraised as a threat (Anshel et al., 2001; Levy et al., 2010). It appears that the context (competition or training) is also a factor of influence for the selection of a coping strategy. Athletes will more likely use a problem- and emotion-focused coping strategy when they experience a stress situation during competition independent of rating of stress intensity.

Previous research has suggested that team athletes are more likely to use emotion and avoidance coping strategies (Nicholls et al., 2005, 2007). The results of the present study found that the team athletes used more problem- and emotion-focused coping strategies to deal with stress. Nicholls et al. (2012) recently found that challenge appraisal was more likely associated with using more adaptive problem-focused coping strategies. However, the different findings between previous studies and the present study could also because of variations in the categorization of the coping strategies (Nicholls and Polman, 2007). This study adopted three higher order coping dimensions and a no coping dimension, whereas Nicholls et al. (2007) included no coping in the avoidance coping dimension. Also, previous studies used quantitative methods while the present research collected data qualitatively.

This is the first study which has demonstrated that the social environment through important others influence coping in sport. A novel finding is that in these situations the athlete positively valued the uncalled contribution of others, which was more likely to result in the use of adaptive problem- or emotion-focussed coping strategies. These findings were corroborated by the data from the interviews. The team athletes mostly felt the influence on their coping through uncalled-for communication and encouragement that was given by their team members. These mainly verbal behaviors of team-mates influenced the way the athletes coped with the stressful encounter. However, they are not part of the actual coping strategies employed to deal with the situation. Importantly, the social environment appears to have resulted in athletes employing more adaptive coping strategies, which ultimately would result in enhanced performance and satisfaction although this would require further research.

The interview data suggests that the influence of others appeared mostly to have an indirect effect on coping of the athletes through its appraisal. It has to be stressed that the influence of others in this study is not the same as social support, which is generally classified as an emotion-focused coping strategy (Nicholls et al., 2007). Hence, if this was the case than it would be expected that stress intensity would have been lower in the diary study. Also, the participants reported the influence they felt from the uncalled behavior of others during the stress and coping process (our definition). This is different from seeking social support, in which the athlete appraises the stressor and uses informational or emotional social support from others as a coping strategy (Nicholls et al., 2007).

A strength of the present research is the use of two different methods to examine the effect of the social environment context through others on the stress and coping of team athletes. The diary study used a homogenous group of field hockey athletes limiting the generalizability of the findings. Therefore, for the interview part of the study athletes from different sports were recruited to ascertain whether the findings of the diary study were applicable to other team sports. Also, the present study cannot conclude whether others are the cause or the effect on the differences in appraisal and the use of coping strategies. It could be that athletes feel the influence of others and therefore will cope with the situation directly by focussing on the task or by dealing with the resulting emotions. However, it is also possible that the use of these coping strategies will cause the team athletes to experience influence of others on their stress and coping. Future research should try to distinguish whether the influence of teammates is causing athletes to choose the selection of a particular coping strategy, or whether it is the use of these coping strategies that allows athletes to experience more influence of the environment through others. Future research could examine the different types of reactions of teammates and which is of most influence on team athlete’s stress and coping process (verbal vs. non-verbal). Knowing which teammate behavior is responsible for behavioral changes in team athletes can be of value to athletes to cope more effectively thereby optimizing performance.

The present research tried to diminish retrospective recall by asking, and frequently reminding participants to complete their diaries as soon as possible after a competition or training. Even though the time between the competition/training and diary completion was reduced there is the possibility for retrospective bias or distortion. Participants’ reports could have been influenced by the way situation was resolved. Finally, when participants reported an influence of the uncalled behavior of others, they could only report on the effect of others on their appraisal of stress and coping of which they were consciously aware. However, this does not rule out the possibility of an influence of others that was not recognized by the participants, and therefore not reported in the diaries or interviews.

Team athletes often experience stress in which their appraisal and coping is influenced by their social environmental context through others (teammates in particular). The intensity of the stress is highest during game situations or when the athletes appraise the situation as a threat. However, others are not of influence on the stress intensity. In challenge situations athletes use more problem- and emotion-focus coping strategies whereas avoidance and no coping is more frequently reported in threat situations. Future studies need to look more closely into which behavior of others is of influence on the stress and coping of team athletes. The present study provides novel findings of the effect of the social environment can have on the stress and coping of team athletes and suggests that others can influence the coping with stress in athletes. This would provide a new portal for sport psychologists or coaches for future interventions.

## Author Contributions

RP research idea, study design, data analysis paper writing. CK Data collection, data analysis, paper writing. JV: Study design, paper writing.

## Conflict of Interest Statement

The authors declare that the research was conducted in the absence of any commercial or financial relationships that could be construed as a potential conflict of interest.
